# Who Uses Services Overall and Within High‐Need Families? Social Inequalities in Access to Early Support Services in Southern Germany

**DOI:** 10.1111/cch.70346

**Published:** 2026-09-27

**Authors:** Sonja Herrmann, Ina Nehring, Volker Mall, Anna Friedmann

**Affiliations:** ^1^ Chair of Social Pediatrics, TUM School of Medicine and Health Technical University of Munich Munich Germany; ^2^ Faculty of Psychology and Educational Sciences Ludwig Maximilian University Munich Germany; ^3^ HMU Health and Medical University Potsdam Germany; ^4^ German Center for Child and Adolescent Health (DZKJ), Munich Partner Site Munich Germany; ^5^ kbo Children's Centre Munich Germany

**Keywords:** early support services, Germany, parenting stress, psychosocial burden, service utilization, social inequalities

## Abstract

**Objectives:**

Reducing inequalities in early childhood support is a central public health and prevention challenge because services are most effective when families with high psychosocial burden can access them. This study examined a potential need–access gap in Early Support Services (Frühe Hilfen) in Southern Germany by analysing overall service use and use within a subgroup of highly burdened parents.

**Materials and Methods:**

Cross‐sectional data from the Junge Familien in Bayern (JuFaBY) cohort of families with young children were used. High need was defined as membership in the highest cut‐off category of the Eltern‐Belastungs‐Inventar (EBI), indicating clinically significant parenting stress. Multivariable logistic regression models with robust standard errors were estimated, adjusting for socio‐economic position, family structure, parental mental health, parenting behaviour difficulties, child health, childcare hours, labour status, survey year and non‐linear child age effects.

**Results:**

In the full sample (*N* = 20 216), Early Support use was positively associated with several need indicators, including parenting stress, parenting behaviour difficulties, single parenthood and chronic child illness. A socio‐economic gradient was also observed: Lower household income was associated with higher uptake, while higher parental education was associated with higher use. In the high‐need subgroup (*N* = 3232), income differences attenuated, but disparities by parental education and migration proxy persisted.

**Conclusion:**

Early Support Services appear broadly need‐responsive, while selective access barriers may remain among families with high support needs. In particular, educational and migration‐related differences in uptake within highly burdened families indicate potential inequalities in access despite comparable levels of need.

## Summary

- Early Support Services showed overall need‐responsive uptake, with higher use among families experiencing several forms of psychosocial burden.
- Higher parental education was associated with greater service use, including within the subgroup of highly stressed parents.
- Among high‐need families, having at least one foreign‐born parent was associated with lower service uptake.
- Access‐sensitive implementation, including proactive outreach and low‐threshold referral pathways, may help reduce inequalities in service uptake.
- Future research should examine service type, timing, duration, frequency, and continuity to capture differences in the ‘dose’ of support received.

## 1 Introduction

Early childhood is a sensitive developmental period in which family stress, parental mental health and caregiving resources can have substantial implications for child well‐being and development (Shonkoff and Garner 2012; Black et al. 2017). In Germany, Early Support Services (Frühe Hilfen) were established as low‐threshold services for families with young children, particularly those in stressful life situations, through coordinated structures across health care, child and youth welfare and related sectors (Nationales Zentrum Frühe Hilfen [NZFH] 2024). From a public health and prevention perspective, a key question is not only whether these services are available, but whether they reach families with elevated support needs (NZFH 2024).

Existing research suggests that the use of Frühe Hilfen is shaped by both need and social position. Families with elevated psychosocial burden may have particularly high support needs, but they may also face barriers to service uptake (Eickhorst et al. 2016). The empirical picture is therefore not one of simple underuse, but of selective uptake shaped by need, socio‐economic resources and access pathways (Eickhorst et al. 2016; Fullerton et al. 2018; Streffing et al. 2022).

Using data from the nationwide *Kinder in Deutschland—KiD 0–3* (Children in Germany 0–3) study, which recruited more than 8000 parents of children aged 0–3 years through paediatric practices across Germany, Eickhorst et al. (2016) showed that psychosocial burden was systematically related to knowledge and use of support services, while uptake differed between universal and more targeted offers. Their findings suggest that universal offers tend to be used more often by socially advantaged families, whereas some targeted early support formats are more likely to reach socially disadvantaged groups, reflecting proactive offering and referral through professionals and institutions (Eickhorst et al. 2016).

Beyond socio‐economic status, the literature suggests that access is also shaped by information pathways, institutional trust, and perceived stigma. A mixed‐methods study on how local authorities establish contact with vulnerable families found that home visits can be effective but may also be perceived as monitoring; earlier, universal information provision and contact via routinely trusted professionals such as midwives, paediatricians and obstetricians were identified as strategies to reduce barriers (Streffing et al. 2022). Complementing this, qualitative work emphasizes that uptake is not fully explained by classic sociodemographic factors alone. Culturally shaped orientations and perceived parental ‘steering competence’ may also influence whether families engage with support, especially in the context of primary preventive services (Neumann and Renner 2016).

Social disparities in access have also been described for migration‐related factors, particularly for universal preventive and health‐related services. An NZFH factsheet based on KiD 0–3 reported lower knowledge of available services and lower use of some health‐care‐related supports among families with migration‐related disadvantages, even after adjustment for socio‐economic status; limited German‐language proficiency was identified as a key barrier (Fullerton et al. 2018). At the same time, the same evidence base suggests that more targeted early support offers, such as family midwife services, may be offered at similar rates and used at comparable levels once proactively offered, underscoring the importance of active outreach and low‐threshold referral pathways (Fullerton et al. 2018; NZFH 2024).

As a heuristic, these findings can be organized using broader access‐to‐services frameworks. Andersen's Behavioural Model of Health Services Use (Andersen 1995) distinguishes between predisposing characteristics, enabling resources and need factors, while Levesque et al. (2013) emphasize the interaction between service accessibility and families' ability to perceive, seek, reach and engage with support. In the present study, parenting stress, depressive symptoms, parenting behaviour difficulties and child chronic illness are treated as indicators of need. Education, income and labour status are modelled as enabling resources, while the migration proxy (any parent foreign‐born) is included as a social‐position‐related indicator that may be associated with access pathways not directly observed in the dataset.

Parenting stress is a central indicator of psychosocial burden in families with young children. Elevated parenting stress has been linked not only to less favourable child outcomes, including emotional and behavioural difficulties, but also to difficulties in everyday parenting, such as lower parental sensitivity and more dysfunctional parenting behaviour (Abidin 1992; Deater‐Deckard 1998). At the same time, high stress may reduce parents' capacity to seek, organize and engage with available support. In the present study, psychological burden parameters therefore include both parental stress and parenting‐related difficulties, alongside parental mental health and child health indicators. Parenting stress is treated as both a continuous indicator of need and the defining criterion for the high‐need subgroup. High need is operationalized as clinically significant parenting stress based on the highest EBI cut‐off category (T ≥ 70).

The present study examines two related questions within one analytic framework. First, we assess which families use Early Support Services in the JuFaBY sample, focusing on psychological burden parameters and sociodemographic factors associated with service uptake. Second, we restrict the analysis to a high‐need subgroup of highly stressed parents and examine which factors are associated with service use within this group. This second step shifts the focus from general utilization patterns to potential within‐need inequalities, that is, whether some highly burdened parents remain less likely to access support than others.

By combining a full‐sample utilization model with a high‐need subgroup analysis, the study distinguishes between overall need sensitivity of the service system and selective access among families with comparably high burden. This design allows a more precise assessment of whether Early Support Services in this sample reflect need‐related reach, selective access disparities, or both.

## 2 Data and Methods

### 2.1 Study Design and Sample

We used cross‐sectional data from the baseline assessment of the JuFaBY cohort. JuFaBY (*Junge Familien in Bayern*) is a Bavaria‐wide follow‐up study to the earlier ‘CoronabaBY’ project, examining how ongoing crisis conditions affect families with young children. The study focuses on parents of children aged 0–9 years and aims to identify current stressors and support needs. Data are collected digitally via the PraxisApp ‘Mein Kinder‐ und Jugendarzt’, through which families are invited to participate. Parents complete questionnaires at two time points spaced 6 months apart, reporting on their own psychosocial burden and their child's well‐being. All responses are transmitted in anonymized form to the research team at the Chair of Social Pediatrics at the Technical University of Munich (Friedmann et al. 2022).

We decided against multiple imputation, as the proportion of item‐level nonresponse was low (see summary statistics in Table A1). Most survey items were mandatory, and incomplete or partially completed questionnaires were not provided by the service provider and are therefore not included in the dataset. The analytic sample for the full model comprised *N* = 20 216 participants with complete information on early support utilization and all covariates (for detailed summary statistics on the full sample, please see Table A1). For analyses restricted to highly stressed parents (high‐need subsample), the sample size was *N* = 3232.

### 2.2 Measures

#### 2.2.1 Early Support Utilization

Utilization of early support services (FH_use) was derived from a multiple‐response item assessing whether parents had ever used any services classified as ‘Frühe Hilfen’ (e.g., KoKi [Koordinierende Kinderschutzstellen, engl.: coordinating child protection centers], family midwife services, counselling centres, early childhood groups, attachment‐based home‐visiting programs). Responses included a range of specified services as well as an option indicating no use of any listed or similar services. A binary variable was constructed (1 = use of at least one service, 0 = no use).

For the high‐need subsample analysis, the same binary indicator was applied within the subgroup of highly stressed parents to examine service utilization among those with elevated support needs.

#### 2.2.2 Parental Stress (EBI)

Parental stress was assessed with the *Eltern‐Belastungs‐Inventar* (EBI), the German version of the Parenting Stress Index (PSI) originally developed by Abidin (1995) and adapted by Tröster (2011). The EBI is a multidimensional parent‐report instrument assessing parental functioning across domains including role restriction, competence, attachment and social isolation. JuFaBY administered the 24‐item parental domain used for all participants, with four additional partner‐relationship items available where applicable; the full 48‐item version also includes a child domain which was not used in this study; a total score reflecting overall parental stress was used in analyses and z‐standardized. The EBI has demonstrated high internal consistency and utility as a screening measure of stress in parents of young children. Internal consistency was excellent in the analytic sample (Cronbach's α = 0.92 for the 24‐item parental domain score; α = 0.93 when including the four partner‐relationship items). For regression analyses, the score was z‐standardized (mean = 0, SD = 1).

Scores were interpreted based on age‐appropriate normative T‐values provided in the manual. In line with the instrument guidelines, T‐scores in the normative range (approximately T < 60) were considered unremarkable, values between about T = 60–69 as elevated and T ≥ 70 as clinically significant parenting stress. For the identification of high‐need parents, we used the upper category (T ≥ 70).

#### 2.2.3 Parental Mental Health and Parenting Behaviour

Depressive symptoms and anxiety were assessed with the Patient Health Questionnaire depression module (PHQ‐4, Cronbach's α = 0.81), and the resulting score was categorized into severity levels for analysis (Kroenke et al. 2009).

Parenting behaviour difficulties were assessed using the hostile‐reactive parenting scale of the Comprehensive Early Childhood Parenting Questionnaire (CECPAQ; Verhoeven et al. 2017). The four‐item scale captures stress‐reactive and mood‐dependent parenting responses. Items were scored such that higher values indicate greater parenting difficulties. Internal consistency in the analytic sample was acceptable to good (Cronbach's α = 0.79). The resulting scale score was z‐standardized (mean = 0, SD = 1) for the regression analyses.

#### 2.2.4 Socio‐Economic Position

Household net income was assessed in six ordered categories (< 1500, 1500–2499, 2500–3499, 3500–4499, 4500–5499, ≥ 5500 EUR) as surveyed, with an additional category for non‐disclosure. The non‐disclosure category was retained as a separate predictor to account for potential systematic differences associated with income non‐response and to reduce bias due to item‐level missingness.

Parental education was assessed via self‐report with seven response options ranging from no school‐leaving certificate to doctorate. For analysis, categories were collapsed into three groups: low (no or lower secondary qualification, including ‘other’), medium (intermediate secondary) and high (university entrance qualification or tertiary degree). The ‘other’ category was assigned to the low group due to small case numbers and limited comparability.

The parental migration proxy was coded as a binary variable, with 0 indicating that both parents were born in Germany and 1 indicating that at least one parent was born outside Germany.

Internal consistency estimates for all multi‐item measures used in the analyses are summarized in Table A5.

### 2.3 Covariates

Models adjusted for single‐parent status (1 = single, 0 = others), child age (in months), child sex (1 = male, 0 = female), presence of chronic child illness or disability (1 = chronic disease or disability, 0 = no disease), weekly childcare hours (2 = more than 20 h, 1 = up to 20 h, 0 = no childcare), parental labour status (dummy‐coded variables: employed, parental leave, in education, unemployed, homemaker, other) and siblings in household (1 = at least one sibling in household, 0 = no siblings). We included year of data collection as a control to account for period‐specific differences in context and fieldwork, thereby reducing bias from temporal heterogeneity (0 = 2023, 1 = 2024).

### 2.4 Statistical Analysis

We estimated multivariable logistic regression models. Model 1 examined predictors of early support utilization in the full sample (*N* = 20 216). Child age was modelled flexibly using restricted cubic splines (natural splines, df = 4) to allow for non‐linear age effects. Model 2 focused on the high‐need subsample (*N* = 3232) and examined which factors were associated with utilization among highly stressed parents. The same set of covariates was included, and child age was again modelled using natural splines (df = 4). Robust standard errors (HC1) were used to account for potential heteroscedasticity. Results are reported as odds ratios (ORs) with 95% confidence intervals and average marginal effects (AMEs, see Appendix A). Model fit was assessed using McFadden's pseudo‐*R* ^2^. Predicted probabilities over child age were derived from the fitted models by holding all other covariates constant at their median (continuous variables) or modal (categorical variables) values.

## 3 Results

In the full sample (Model 1; see Figure 1 and Table A2), uptake of Early Support Services (FH use) was associated with several indicators of psychosocial burden and family context. Higher parenting stress (EBI, z‐standardized) was associated with higher odds of service use (OR = 1.185, *p* < 0.001), corresponding to a modest average marginal increase of 4.02 percentage points in predicted use per 1 SD increase. Greater parenting behaviour difficulties (CEC; z‐standardized) were associated with higher odds of service use (OR = 1.072, *p* < 0.001), corresponding to an average marginal effect of +1.65 percentage points. By contrast, depressive symptoms and anxiety (PHQ categories) were not clearly associated with uptake after adjustment (OR = 1.063, *p* > 0.05; OR = 0.917, *p* > 0.05).

**FIGURE 1:**
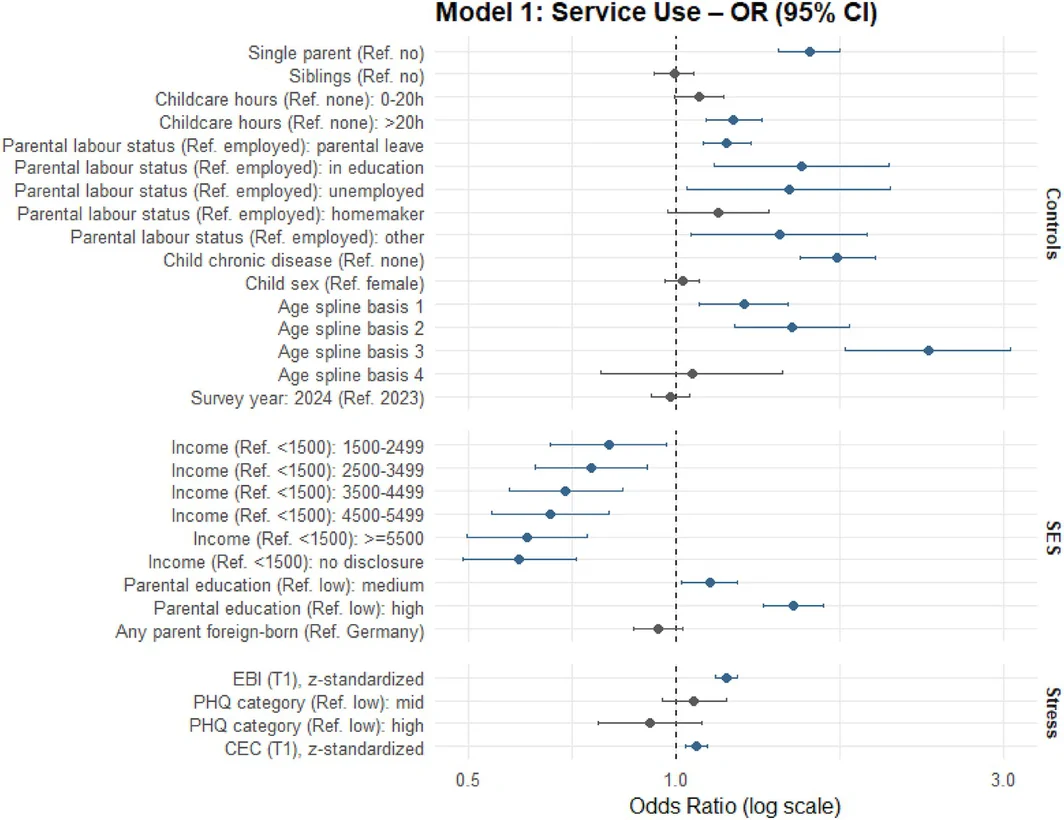
Adjusted odds ratios for Early Support use in the full sample (Model 1, *N* = 20 216).

A socio‐economic pattern was evident. Compared with the lowest income category, all higher income categories (including non‐disclosure) showed lower odds of use. Medium (OR = 1.12, *p* < 0.05) and high parental education (OR = 1.48, *p* < 0.001) were associated with higher odds of service use. In probability terms, this corresponded to average marginal effects of +2.62 percentage points for medium education and +9.27 percentage points for high education, relative to low parental education. The migration proxy (any parent foreign‐born) was not statistically associated with uptake in the full‐sample model (OR = 0.941, *p* > 0.05). In addition, single parenthood (OR = 1.564, *p* < 0.001), chronic child disease (OR = 1.716, *p* < 0.001), more than 20 h of childcare (OR = 1.214, *p* < 0.001) and most parental labour‐status categories (relative to employed) were associated with higher odds of use. Child age showed a non‐linear association (modelled with splines), while child sex (OR = 1.021, *p* > 0.05), siblings (OR = 0.993, *p* > 0.05) and survey year (OR = 0.982, *p* > 0.05) were not clearly associated with uptake.

For the full‐sample model (Model 1), the predicted probability of using Early Support Services varied non‐linearly across child age (Figure 2). The curve shows a relatively steep increase during the first months of life, followed by a dip in the second/third year and then a renewed increase towards later ages before flattening slightly at the upper end of the observed age range. Overall, this indicates that service use is age‐dependent and not well represented by a simple linear term. Uncertainty is wider at the end of the age distribution, so the shape at the oldest ages should be interpreted more cautiously.

**FIGURE 2:**
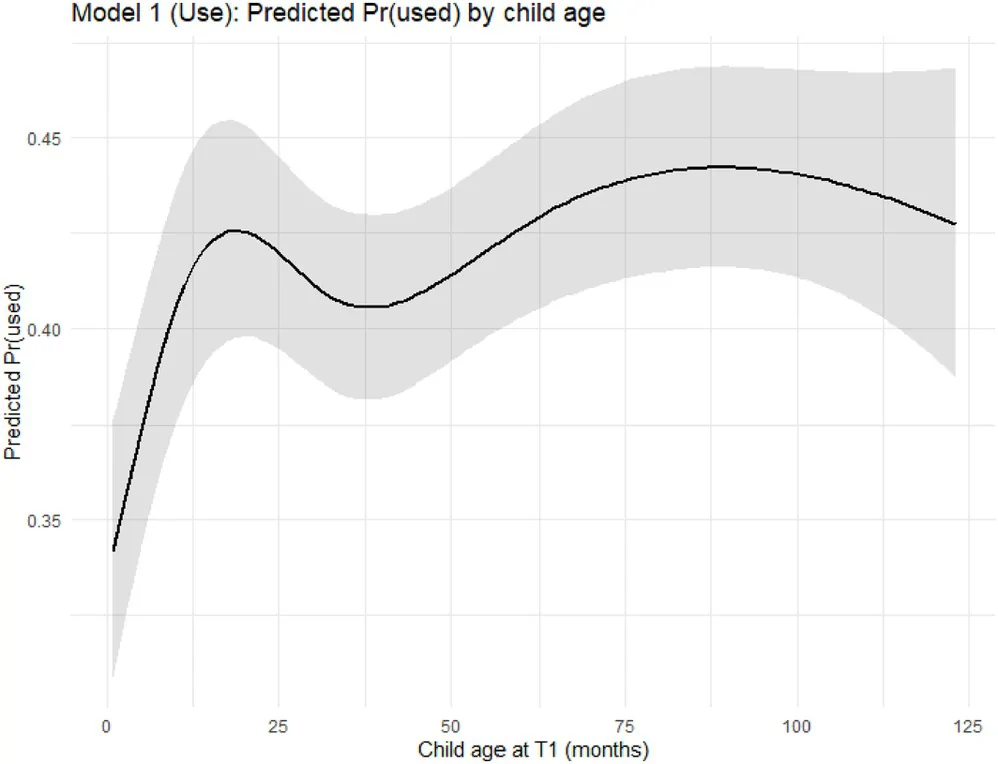
Predicted probability of Early Support use by child age (Model 1).

In the high‐need subgroup (Model 2, see Figure 3 and Table A3), the pattern differed: Income categories were not clearly associated with use, whereas high parental education remained positively associated with uptake of Early Support Services (OR = 1.589, *p* < 0.001). In probability terms, the average marginal effect for medium parental education was +3.26 percentage points relative to low parental education and was not statistically clear, whereas high parental education was associated with an average marginal effect of +11.14 percentage points relative to low parental education. In contrast to Model 1, the migration proxy (any parent foreign‐born) was associated with lower odds of use in the high‐need subgroup (OR = 0.724, *p* < 0.01), corresponding to an average marginal difference of −7.78 percentage points in predicted uptake. Parenting stress (EBI) was retained in this restricted model as a continuous covariate to adjust for residual variation in stress severity within the high‐need range. As expected, EBI was not clearly associated with uptake within this subgroup (OR = 1.055, *p* > 0.05). Depressive symptoms and anxiety (PHQ) were again not clearly associated with use (OR = 1.037, *p* > 0.05; OR = 0.91, *p* > 0.05). Parenting behaviour difficulties (CEC) showed only a weak positive association (OR = 1.08, *p* < 0.1). Chronic child disease (OR = 1.801, *p* < 0.001) and parental leave (OR = 1.256, *p* < 0.05) remained positively associated with use, and child age again showed a pronounced non‐linear association. Survey year (2024 vs. 2023) was associated with slightly lower odds of use in this subgroup model (OR = 0.85, *p* < 0.05).

**FIGURE 3:**
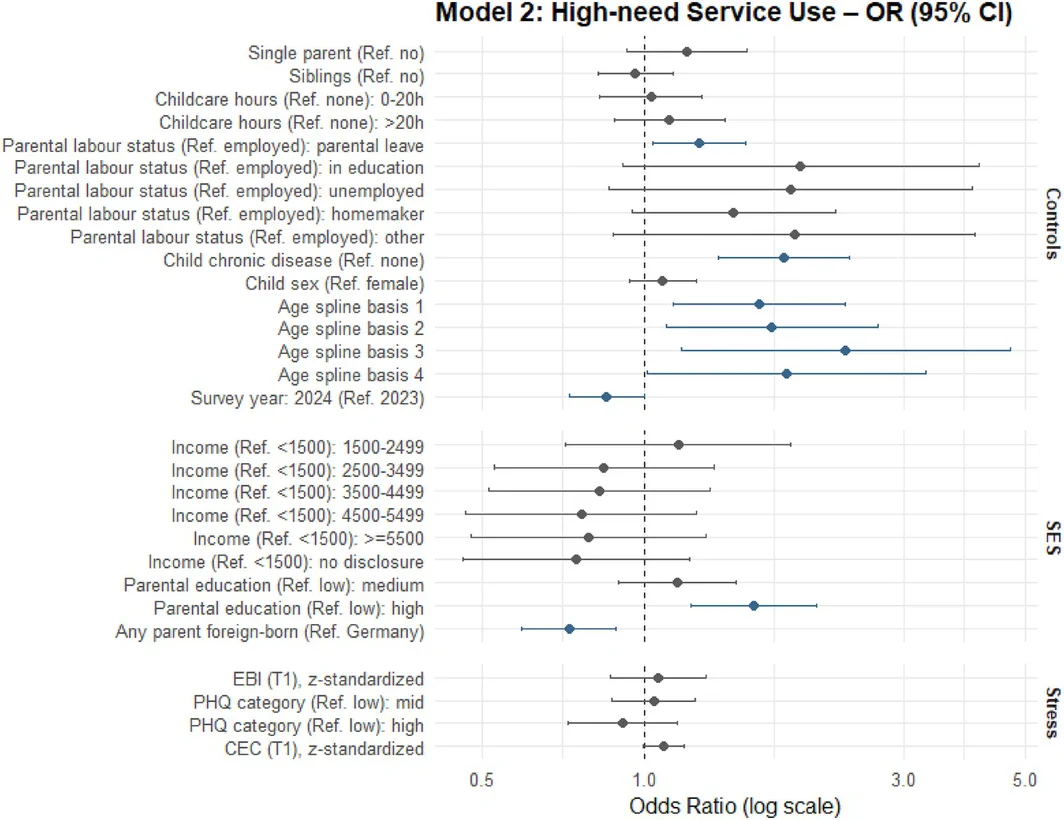
Adjusted odds ratios for Early Support use in the high‐need subgroup (Model 2, *N* = 3232).

In the high‐need subgroup (Model 2), predicted service use increased monotonically with child age, with a steeper rise in the early years and a gradual flattening at older ages (Figure 4). Unlike the full‐sample model, the age pattern was smooth and did not show a mid‐age dip. Confidence intervals widened towards the upper end of the age range, indicating lower precision at the margins.

**FIGURE 4:**
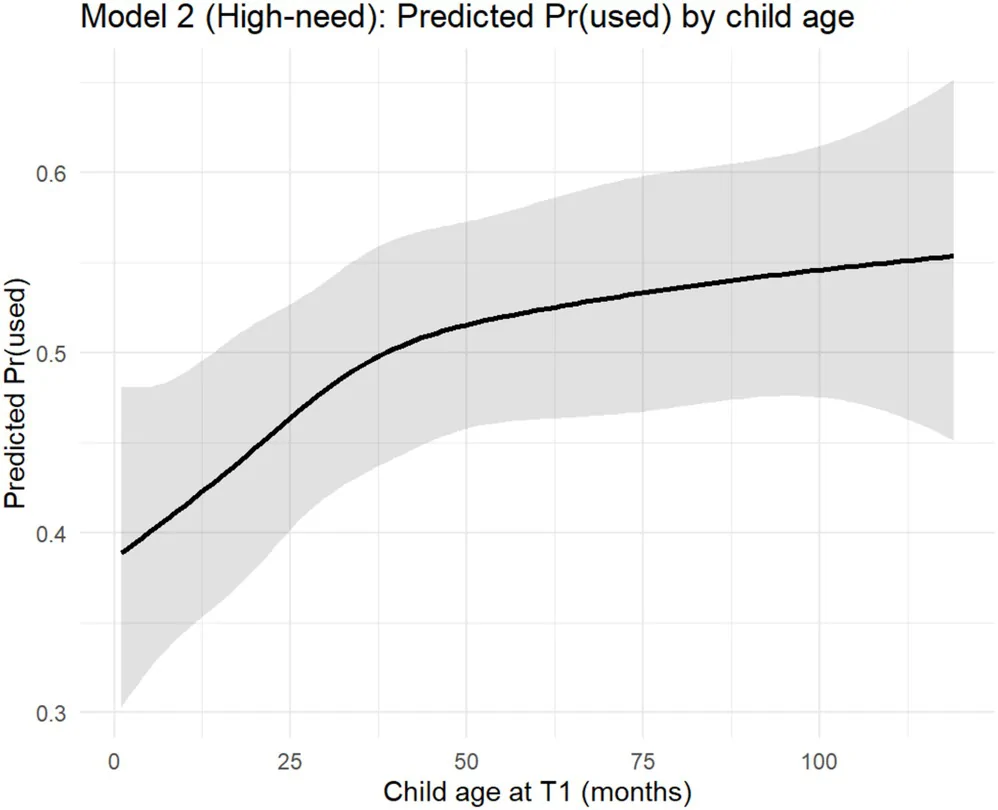
Predicted probability of Early Support use by child age (Model 2, high need).

The interaction model (Model 3; parental education × EBI) was estimated as a supplementary check. The substantive pattern of the main effects was essentially unchanged relative to Model 2, and the interaction terms between parental education and parental stress (EBI) were not statistically significant (OR = 1.112, *p* > 0.05 and OR = 0.892, *p* > 0.05). This model is therefore reported only briefly in Appendix A (see Table A4) and not used for substantive interpretation.

## 4 Discussion

This study examined a potential need–access gap in Bavaria's Early Support Services (Frühe Hilfen) by combining two analytic perspectives: predictors of service use in the full sample and predictors of service use within a subgroup of highly stressed parents. Taken together, the findings suggest a differentiated pattern: On the one hand, the full‐sample model indicates that Early Support Services are responsive to burden‐related need. Higher parenting stress (EBI) and greater parenting behaviour difficulties (CEC) were associated with a higher likelihood of service use, alongside single parenthood and chronic child illness. Depressive symptom burden was included as an indicator of parental mental health but was not clearly associated with service use after adjustment. This pattern is broadly consistent with prior research showing that the use of support services is shaped both by psychosocial burden and by social position (e.g., Eickhorst et al. 2016; NZFH 2024). In the present study, higher parenting stress and greater parenting behaviour difficulties were associated with higher odds of service use in the full sample, suggesting a need‐related pattern of uptake. At the same time, higher parental education was also associated with greater use, indicating that service uptake may additionally depend on social and informational resources. On the other hand, the high‐need subgroup analysis indicates that access is not uniform among highly burdened parents. Within this group, educational disparities persisted, with higher utilization among parents with higher education. In addition, a migration‐proxy‐related disparity became visible that was not apparent in the full‐sample model, with lower utilization among families in which at least one parent was foreign‐born, even after adjustment for multiple covariates. These findings suggest that need sensitivity and equitable access are not identical and are consistent with prior work on the prevention dilemma, as well as evidence that access pathways, informational resources, and migration‐related barriers can shape uptake even in low‐threshold support systems (e.g., Fullerton et al. 2018; Neumann and Renner 2016; Streffing et al. 2022).

Conceptually, the findings map well onto Andersen's distinction between need, enabling resources, and social‐position characteristics (Andersen 1995). In our models, psychosocial burden indicators were included to capture need‐related drivers of utilization, with evidence for need‐related responsiveness particularly in the full‐sample model, whereas education and income are more consistent with enabling resources that shape families' ability to translate need into actual uptake. The emergence of a migration‐related disparity in the high‐need subgroup is also compatible with Levesque et al.'s access framework, which highlights that utilization depends not only on need but on the interaction between service accessibility and users' abilities to perceive, seek, reach, and engage (Levesque et al. 2013). Although our migration proxy captures only a comparatively selective segment of families with migration‐related backgrounds, the observed pattern suggests that inequalities in uptake may persist even where more pronounced language‐related barriers are likely underrepresented. In this sense, the present results suggest that Early Support Services may be need‐responsive at the system level while still showing inequalities at the level of access processes.

A central contribution of the study is therefore conceptual as well as empirical. Distinguishing between who uses services overall and who uses services among those with high need yields a more precise picture of system performance. In the full sample, the association of lower household income with higher service use may be interpreted as evidence of need‐based reach or successful targeting towards families in more disadvantaged circumstances. At the same time, the positive association of higher parental education with service use points to a parallel social gradient in service uptake, likely reflecting differences in information access, navigation skills, trust in institutions, or capacity to engage with available offers. The attenuation of income differences in the high‐need subgroup, alongside persistent educational and migration‐related differences, is particularly informative: once parents are already in a high‐stress category, material differences in income may matter less for uptake than social and informational resources that facilitate access to services.

Within the high‐need subgroup, the absence of a clear association between EBI and service use is not unexpected and should not be overinterpreted as evidence that stress severity is irrelevant. By design, the subgroup was restricted to parents already classified as highly stressed based on the highest EBI cut‐off category. This restriction reduces variability in the underlying construct and shifts the analytic focus from need identification to differential access among families with comparably high burden. In this context, the non‐significant EBI slope is substantively compatible with the study's logic: once high need is established, social and structural factors may play a larger role in shaping who actually accesses services.

The supplementary interaction model (education × EBI) did not provide evidence that the association between parenting stress and service use differs systematically by parental education in the high‐need subgroup. Given that this model was included as a formal sensitivity check and not as a central hypothesis test, the null interaction result should mainly be interpreted as indicating that the main pattern is not driven by a strong education‐specific moderation of the EBI slope. At the same time, the absence of statistical significance does not exclude smaller interaction effects, particularly in subgroup analyses where precision is lower than in the full sample.

From a practice and policy perspective, the present findings suggest that improving need‐based targeting alone may not be sufficient. Intervention research provides some support for access‐sensitive implementation strategies. In the German Early Support context, the PATH intervention, which strengthens referral pathways between paediatricians and Early Support Services, increased families' likelihood of being informed about available services and showed descriptively higher subsequent service use (Glattacker et al. 2026). Experimental evidence from maternal and infant home‐visiting services further suggests that intensified and persistent outreach can increase both successful contact and program enrolment (Jacob et al. 2024). For families with migration‐related backgrounds, evidence is less intervention‐based, but research consistently identifies language‐concordant communication, skilled interpretation, translated information, and culturally responsive professionals as facilitators of service access (Wang and McLaren 2026). Taken together, these findings support proactive outreach, low‐threshold referral pathways, and stronger linkage through trusted professionals, while the effectiveness of specific multilingual and culturally responsive implementation strategies in Early Support Services requires further evaluation. Future work that combines survey data with information on local service infrastructure could help clarify which implementation features are associated with narrower need‐access gaps.

### 4.1 Strengths and Limitations

Several limitations should be considered. First, service use was operationalized as a binary indicator of any use versus no use. This approach is appropriate for a first analysis of reach but does not capture intensity, duration, timing, or type of service use, all of which may differ meaningfully across families. Second, although the models adjust for a broad set of covariates, residual confounding remains possible, especially for variables related to local service infrastructure, referral practices, language accessibility, and institutional trust. Third, complete‐case analysis was used. Item‐level missingness was low and the survey design reduced partial‐response problems, but some selection due to complete‐case restriction cannot be ruled out. Fourth, the sample may be affected by self‐selection due to voluntary participation via an app‐based recruitment pathway. Although this mode of recruitment enabled large‐scale data collection, participating families may differ from non‐participants in unobserved ways related to help‐seeking, institutional trust, or digital access. This may limit generalizability, especially for conclusions about absolute utilization levels. Fifth, the migration proxy variable warrants particular caution. Because participation required sufficient German‐language literacy to complete a digital questionnaire, families with acute language barriers or limited digital access are structurally underrepresented. The migration proxy in this sample therefore likely captures a comparatively selected subgroup and should not be interpreted as representing migration‐related disadvantage in its full range. The binary operationalization (any parent foreign‐born) also conflates diverse migration histories and cannot capture language proficiency, time since migration, or country of origin. Finally, from the perspective of Andersen's model and Levesque et al.'s access framework, several access mechanisms were not directly measured in the present dataset (e.g., knowledge of services, referral pathways, language accessibility, perceived acceptability, and trust in institutions) (Andersen 1995; Levesque et al. 2013). As a result, the observed education‐ and migration‐related disparities should be interpreted as markers of differential access conditions rather than direct tests of specific access mechanisms.

Several strengths merit acknowledgement. First, the large sample (*N* = 20 216 full sample; *N* = 3232 high‐need subgroup) enables stable estimation of subgroup differences. Second, the two‐model design distinguishes between system‐level need sensitivity and within‐need access inequalities, a conceptual contribution not common in Early Support Services research. Third, the use of a validated parenting stress measure (EBI) both as a continuous predictor and as a subgroup‐defining criterion strengthens construct validity. Fourth, robust standard errors, restricted cubic splines for age, and reporting of average marginal effects enhance methodological transparency. Fifth, the concurrent adjustment for psychosocial, socio‐economic, and family structure variables provides a more comprehensive predictor picture than single‐domain approaches.

## 5 Outlook and Further Research

The present findings suggest that Early Support Services show a degree of need responsiveness at the population level, as families with higher psychosocial burden were generally more likely to report service use. At the same time, the results indicate that this responsiveness does not eliminate social inequalities in access. In particular, within the subgroup of highly burdened families, disparities by parental education and migration‐related background remained visible, pointing to a persisting need–access gap even where objective need is high. Against this background, the next step is not only to examine who accesses services, but also how use unfolds over time and whether it is associated with changes in parental and child well‐being.

The next analytic step is to use the longitudinal JuFaBY data and the ongoing follow‐up project Junge Familien in Deutschland (JuFaDe) to move from cross‐sectional utilization patterns towards trajectories and consequences of service use. Several directions are especially promising: First, uptake at T1 can be linked to changes in parental stress, anxiety and depressive symptoms, and child outcomes at T2, with explicit attention to baseline burden and selection into service use. This would allow a more direct examination of whether Early Support use is associated with short‐term improvements and for whom. Second, timing and persistence of stress could be modelled more explicitly (e.g., stable high burden vs. remission/new onset profiles), which would align well with the present ‘need–access gap’ framework and extend it from access to potential effectiveness under real‐world conditions. Third, future analyses should differentiate service types (e.g., universal vs. outreach‐oriented or targeted services) where possible, since the mechanisms of access and social selectivity are likely to differ across formats. Fourth, future studies should move beyond binary indicators of service uptake and examine the ‘dose’ of Early Support Services, including frequency, duration, timing, and continuity of use. This would allow analyses of whether social inequalities extend beyond initial access to differences in the intensity and persistence of service engagement and whether service dose is associated with subsequent parental and child outcomes.

## Author Contributions

**Sonja Herrmann:** conceptualization, methodology, investigation, formal analysis, visualization, writing – review and editing, writing – original draft, data curation. **Ina Nehring:** writing – review and editing. **Volker Mall:** writing – review and editing. **Anna Friedmann:** writing – review and editing, funding acquisition, supervision.

## Funding

The authors have nothing to report.

## Ethics Statement

The JuFaBY study protocol was approved by the Ethics Committee of the Technical University of Munich (vote no. 2022‐483_1‐S‐KH). The study was preregistered in the Open Science Framework (OSF) (https://osf.io/8aj6k/overview).

## Consent

Informed consent was obtained from all individual participants included in the study.

## Conflicts of Interest

The authors declare no conflicts of interest.

## Data Availability

The data are not publicly available due to privacy and ethical restrictions.

## Appendix A

**TABLE A1:** Summary statistics full sample.

Variable	*N* total	Miss (%)	Mean	SD	Median	Min	Max
Fruehe Hilfen use (any use)	20 806	2.46	0.43	0.50	0.00	0.00	1.00
Parenting stress (EBI z‐std)	20 806	0.19	−0.00	1.00	0.05	−2.70	2.95
Parenting behaviour difficulties (CEC z‐std)	20 806	0.20	−0.00	1.00	0.09	−1.94	3.15
Parent foreign born	20 806	0.17	0.15	0.35	0.00	0.00	1.00
Single‐parent household	20 806	0.17	0.10	0.29	0.00	0.00	1.00
Child age (months)	20 806	0.16	41.23	34.31	32.00	0.00	151.00
Child sex	20 806	0.53	0.52	0.50	1.00	0.00	1.00
Child chronic disease	20 806	0.17	0.06	0.23	0.00	0.00	1.00
Siblings in household	20 806	0.17	0.57	0.50	1.00	0.00	1.00

Variable	*N* total	Miss (%)	Level	*n* (%)
Depressive symptoms and anxiety (PHQ‐4)	20 806	0.19	Low	18 480 (89.0%)
			Mid	1676 (8.1%)
			High	610 (2.9%)
Household net income	20 806	0.17	< 1500	590 (2.8%)
			1500–2499	1997 (9.6%)
			2500–3499	3607 (17.4%)
			3500–4499	4438 (21.4%)
			4500–5499	3151 (15.2%)
			≥ 5500	2883 (13.9%)
			No disclosure	4105 (19.8%)
Parental education	20 806	0.17	Low_other	2592 (12.5%)
			Medium	10 250 (49.3%)
			High	7929 (38.2%)
Childcare hours	20 806	0.17	None	6674 (32.1%)
			0–20 h	6576 (31.7%)
			> 20 h	7521 (36.2%)
Parental labour status	20 806	0.18	Employed	11 904 (57.3%)
			Parental_leave	7702 (37.1%)
			In_education	195 (0.9%)
			Unemployed	145 (0.7%)
			Homemaker	633 (3.0%)
			Other	190 (0.9%)
Survey year	20 806	0.16	2023	15 154 (73.0%)
			2024	5619 (27.0%)

### Regression Results (Robust OR/AME Tables)

**TABLE A2:** Model 1: FH use (full sample).

Term	OR (95% CI)	AME	*p*	Sig
(Intercept)	0.518 (0.414, 0.647)		0.0000	***
Income (ref. < 1500) × 1500–2499	0.798 (0.657, 0.968)	−5.45 [−10.12, −0.78]	0.0223	*
Income (ref. < 1500) × 2500–3499	0.753 (0.623, 0.909)	−6.85 [−11.39, −2.30]	0.0032	**
Income (ref. < 1500) × 3500–4499	0.692 (0.572, 0.836)	−8.87 [−13.43, −4.31]	0.0001	***
Income (ref. < 1500) × 4500–5499	0.657 (0.54, 0.799)	−10.10 [−14.81, −5.39]	0.0000	***
Income (ref. < 1500) × ≥ 5500	0.608 (0.498, 0.743)	−11.89 [−16.69, −7.10]	0.0000	***
Income (ref. < 1500) × no disclosure	0.591 (0.489, 0.715)	−12.55 [−17.10, −8.00]	0.0000	***
Parental edu (ref. low) × medium	1.12 (1.019, 1.23)	2.62 [0.46, 4.78]	0.0184	*
Parental edu (ref. low) × high	1.48 (1.337, 1.639)	9.27 [6.91, 11.62]	0.0000	***
Any parent foreign‐born (ref. Germany)	0.941 (0.867, 1.021)	−1.43 [−3.35, 0.49]	0.1447	
EBI (T1) z‐standardized	1.185 (1.144, 1.228)	4.02 [3.18, 4.85]	0.0000	***
PHQ cat (ref. low) × mid	1.063 (0.954, 1.185)	1.46 [−1.13, 4.04]	0.2677	
PHQ cat (ref. low) × high	0.917 (0.77, 1.091)	−2.05 [−6.10, 2.01]	0.3267	
CEC (T1) z‐standardized	1.072 (1.034, 1.112)	1.65 [0.80, 2.51]	0.0001	***
Single parent (ref. no)	1.564 (1.41, 1.734)	10.76 [8.27, 13.26]	0.0000	***
Siblings (ref. no)	0.993 (0.93, 1.061)	−0.17 [−1.72, 1.39]	0.8352	
Childcare hours (ref. no childcare) × 0–20 h	1.081 (0.997, 1.173)	1.84 [−0.08, 3.75]	0.0602	.
Childcare hours (ref. no childcare) × > 20 h	1.214 (1.107, 1.332)	4.60 [2.41, 6.79]	0.0000	***
Parental labour (ref. employed) × parental_leave	1.187 (1.096, 1.284)	4.04 [2.18, 5.91]	0.0000	***
Parental labour (ref. employed) × in_education	1.523 (1.137, 2.04)	10.03 [3.01, 17.05]	0.0048	**
Parental labour (ref. employed) × unemployed	1.458 (1.038, 2.047)	8.98 [0.83, 17.14]	0.0295	*
Parental labour (ref. employed) × homemaker	1.152 (0.973, 1.363)	3.34 [−0.66, 7.34]	0.0996	.
Parental labour (ref. employed) × other	1.414 (1.052, 1.899)	8.24 [1.14, 15.33]	0.0216	*
Child chronic disease (ref. none)	1.716 (1.514, 1.945)	13.00 [10.00, 15.99]	0.0000	***
Child sex (ref. female)	1.021 (0.965, 1.081)	0.49 [−0.85, 1.84]	0.4718	
Age spline basis 1	1.254 (1.08, 1.456)	5.35 [1.82, 8.88]	0.0030	**
Age spline basis 2	1.475 (1.219, 1.783)	9.19 [4.70, 13.68]	0.0001	***
Age spline basis 3	2.325 (1.766, 3.06)	19.96 [13.48, 26.43]	0.0000	***
Age spline basis 4	1.054 (0.779, 1.427)	1.24 [−5.92, 8.41]	0.7339	
Survey year (ref. 2023)	0.982 (0.921, 1.047)	−0.43 [−1.95, 1.09]	0.5827	
Pseudo *R* ^2^ (McFadden)			0.0272	
*N*	20 216			

* *p* < 0.05.

** *p* < 0.01.

*** *p* < 0.001.

**TABLE A3:** Model 2: High‐need subgroup (spline model).

Term	OR (95% CI)	AME	*p*	Sig
(Intercept)	0.503 (0.268, 0.946)		0.0329	*
Income (ref. < 1500) × 1500–2499	1.15 (0.713, 1.856)	3.31 [−8.07, 14.70]	0.5665	
Income (ref. < 1500) × 2500–3499	0.84 (0.528, 1.337)	−4.20 [−15.31, 6.91]	0.4613	
Income (ref. < 1500) × 3500–4499	0.825 (0.516, 1.318)	−4.64 [−15.83, 6.56]	0.4200	
Income (ref. < 1500) × 4500–5499	0.764 (0.469, 1.244)	−6.49 [−18.16, 5.17]	0.2786	
Income (ref. < 1500) × ≥ 5500	0.789 (0.48, 1.298)	−5.71 [−17.61, 6.19]	0.3502	
Income (ref. < 1500) × no disclosure	0.746 (0.461, 1.207)	−7.05 [−18.54, 4.45]	0.2331	
Parental edu (ref. low) × medium	1.145 (0.892, 1.471)	3.26 [−2.73, 9.24]	0.2884	
Parental edu (ref. low) × high	1.589 (1.217, 2.074)	11.14 [4.81, 17.47]	0.0007	***
Any parent foreign‐born (ref. Germany)	0.724 (0.594, 0.883)	−7.78 [−12.53, −3.04]	0.0014	**
EBI (T1) z‐standardized	1.055 (0.862, 1.292)	1.30 [−3.58, 6.18]	0.6013	
PHQ cat (ref. low) × mid	1.037 (0.869, 1.236)	0.87 [−3.37, 5.11]	0.6882	
PHQ cat (ref. low) × high	0.91 (0.721, 1.149)	−2.26 [−7.88, 3.35]	0.4295	
CEC (T1) z‐standardized	1.08 (0.991, 1.177)	1.85 [−0.22, 3.93]	0.0809	.
Single parent (ref. no)	1.193 (0.923, 1.541)	4.24 [−1.90, 10.38]	0.1777	
Siblings (ref. no)	0.96 (0.819, 1.126)	−0.98 [−4.82, 2.86]	0.6162	
Childcare hours (ref. no childcare) × 0–20 h	1.026 (0.826, 1.275)	0.62 [−4.63, 5.86]	0.8173	
Childcare hours (ref. no childcare) × > 20 h	1.11 (0.88, 1.4)	2.53 [−3.08, 8.13]	0.3770	
Parental labour (ref. employed) × parental_leave	1.256 (1.032, 1.529)	5.48 [0.80, 10.15]	0.0232	*
Parental labour (ref. employed) × in_education	1.933 (0.909, 4.107)	15.49 [−1.29, 32.28]	0.0867	.
Parental labour (ref. employed) × unemployed	1.856 (0.86, 4.005)	14.59 [−2.71, 31.89]	0.1151	
Parental labour (ref. employed) × homemaker	1.457 (0.945, 2.247)	9.01 [−1.19, 19.22]	0.0883	.
Parental labour (ref. employed) × other	1.884 (0.875, 4.057)	14.93 [−2.25, 32.11]	0.1053	
Child chronic disease (ref. none)	1.801 (1.361, 2.382)	13.93 [7.60, 20.27]	0.0000	***
Child sex (ref. female)	1.077 (0.935, 1.241)	1.79 [−1.63, 5.21]	0.3043	
Age spline basis 1	1.621 (1.126, 2.335)	11.65 [2.89, 20.41]	0.0094	**
Age spline basis 2	1.712 (1.094, 2.679)	12.97 [2.21, 23.73]	0.0185	*
Age spline basis 3	2.341 (1.168, 4.695)	20.51 [3.80, 37.22]	0.0165	*
Age spline basis 4	1.823 (1.011, 3.287)	14.48 [0.30, 28.66]	0.0459	*
Survey year (ref. 2023)	0.85 (0.724, 0.997)	−3.93 [−7.78, −0.09]	0.0453	*
Pseudo *R* ^2^ (McFadden)			0.0258	
*N*	3232			

* *p* < 0.05.

** *p* < 0.01.

*** *p* < 0.001.

**TABLE A4:** Model 3: High‐need subgroup (interaction model).

Term	OR (95% CI)	AME	*p*	Sig
(Intercept)	0.505 (0.185, 1.38)		0.1829	
Income (ref. < 1500) × 1500–2499	1.147 (0.705, 1.864)	3.24 [−8.14, 14.61]	0.5811	
Income (ref. < 1500) × 2500–3499	0.837 (0.522, 1.341)	−4.29 [−15.38, 6.80]	0.4586	
Income (ref. < 1500) × 3500–4499	0.824 (0.512, 1.326)	−4.64 [−15.82, 6.53]	0.4256	
Income (ref. < 1500) × 4500–5499	0.762 (0.465, 1.25)	−6.53 [−18.18, 5.12]	0.2825	
Income (ref. < 1500) × ≥ 5500	0.786 (0.475, 1.302)	−5.78 [−17.67, 6.10]	0.3504	
Income (ref. < 1500) × no disclosure	0.745 (0.458, 1.214)	−7.07 [−18.55, 4.41]	0.2379	
Parental edu (ref. low) × medium	0.981 (0.375, 2.565)	3.27 [−2.72, 9.25]	0.9681	
Parental edu (ref. low) × high	1.879 (0.709, 4.983)	11.14 [4.81, 17.47]	0.2048	
Any parent foreign‐born (ref. Germany)	0.722 (0.592, 0.882)	−7.84 [−12.58, −3.10]	0.0014	**
EBI (T1) z‐standardized	1.056 (0.596, 1.872)	1.34 [−3.53, 6.20]	0.8512	
PHQ cat (ref. low) × mid	1.037 (0.869, 1.238)	0.89 [−3.36, 5.13]	0.6837	
PHQ cat (ref. low) × high	0.909 (0.719, 1.149)	−2.31 [−7.92, 3.30]	0.4229	
CEC (T1) z‐standardized	1.08 (0.991, 1.178)	1.86 [−0.21, 3.94]	0.0807	.
Single parent (ref. no)	1.19 (0.919, 1.542)	4.19 [−1.95, 10.33]	0.1868	
Siblings (ref. no)	0.957 (0.815, 1.124)	−1.05 [−4.90, 2.79]	0.5944	
Childcare hours (ref. no childcare) × 0–20 h	1.023 (0.822, 1.273)	0.55 [−4.69, 5.80]	0.8376	
Childcare hours (ref. no childcare) × > 20 h	1.11 (0.879, 1.403)	2.53 [−3.08, 8.14]	0.3802	
Parental labour (ref. employed) × parental_leave	1.257 (1.031, 1.531)	5.49 [0.82, 10.17]	0.0234	*
Parental labour (ref. employed) × in_education	1.943 (0.89, 4.243)	15.61 [−1.14, 32.35]	0.0956	.
Parental labour (ref. employed) × unemployed	1.867 (0.836, 4.168)	14.72 [−2.58, 32.02]	0.1275	
Parental labour (ref. employed) × homemaker	1.45 (0.932, 2.256)	8.90 [−1.37, 19.16]	0.0990	.
Parental labour (ref. employed) × other	1.861 (0.845, 4.096)	14.64 [−2.55, 31.83]	0.1229	
Child chronic disease (ref. none)	1.796 (1.355, 2.382)	13.87 [7.53, 20.21]	0.0000	***
Child sex (ref. female)	1.074 (0.931, 1.239)	1.73 [−1.70, 5.15]	0.3254	
Age spline basis 1	1.625 (1.126, 2.347)	11.71 [2.94, 20.47]	0.0096	**
Age spline basis 2	1.72 (1.095, 2.702)	13.08 [2.31, 23.84]	0.0185	*
Age spline basis 3	2.361 (1.172, 4.758)	20.71 [3.99, 37.42]	0.0162	*
Age spline basis 4	1.83 (1.009, 3.32)	14.57 [0.40, 28.74]	0.0466	*
Survey year (ref. 2023)	0.847 (0.721, 0.994)	−4.01 [−7.86, −0.17]	0.0423	*
Parental edu (ref. low) × medium: EBI_z	1.112 (0.59, 2.095)	2.55 [−12.48, 17.58]	0.7437	
Parental edu (ref. low) × high: EBI_z	0.892 (0.471, 1.691)	−2.75 [−17.91, 12.41]	0.7265	
Pseudo *R* ^2^ (McFadden)			0.0260	
*N*	3232			

* *p* < 0.05.

** *p* < 0.01.

*** *p* < 0.001.

**TABLE A5:** Reliability results.

Measure	Items	*N*	Cronbach's α
EBI parental domain, excluding partner relationship items	24	20 216	0.92
EBI parental domain, including partner relationship items	28	19 511	0.93
PHQ‐4 total	4	20 216	0.81
CECPAQ hostile‐reactive parenting	4	20 216	0.79
